# Serological Evidence of Potential Marburg Virus Circulation in Livestock and Dogs in Ghana

**DOI:** 10.3390/pathogens13110917

**Published:** 2024-10-22

**Authors:** Theophilus Odoom, Sherry Ama Mawuko Johnson, William Tasiame, Meyir Y. Ziekah, Joseph K. Abuh, Benita Anderson, Fenteng Danso, Richard K. Abbiw, Franklin Y. Nuokpem, Emmanuel Allegye-Cudjoe, Charles Lewis, Bonto Faburay

**Affiliations:** 1 Veterinary Services Department, Ministry of Food and Agriculture, Accra M-37, Ghana; josephabuh@gmail.com (J.K.A.); fentengdanso6@gmail.com (F.D.); emmallec@yahoo.com (E.A.-C.); 2 School of Veterinary Medicine, University of Ghana, Accra LG-25, Ghana; amada61112@gmail.com; 3 Department of Veterinary Public Health and Epidemiology, School of Veterinary Medicine, Kwame Nkrumah University of Science and Technology, Kumasi 00233, Ghana; drwilly2002@gmail.com; 4 Kumasi Zoological Gardens, Wildlife Division, Forestry Commission, Kumasi AK-181-1074, Ghana; meyir73@gmail.com; 5 West African Centre for Cell Biology of Infectious Pathogens, University of Ghana, Accra LG-25, Ghana; abbiw.richardk@gmail.com (R.K.A.); fynuokpem@gmail.com (F.Y.N.); 6 Foreign Animal Disease Diagnostic Laboratory, National Bio and Agrodefense Facility, Animal and Plant Health Inspection Service, United States Department of Agriculture, Manhattan, KS 66506, USA; charles.lewis@usda.gov (C.L.); bonto.faburay@usda.gov (B.F.)

**Keywords:** emerging zoonosis, Marburg virus antibodies, Ghana, Marburg exposure, MARV, livestock

## Abstract

Marburg virus disease (MVD) is a zoonotic hemorrhagic disease with an estimated case fatality rate of up to 88%. Ghana recorded its first human MVD outbreak in June 2022 and although the outbreak was quickly brought under control, the transmission dynamics of the disease remained unclear. We assessed the presence of Marburg virus (MARV) antibodies in livestock and dogs and identified associated risk factors that increased the risk of these animals being exposed to MARV in five regions of Ghana. Sera collected from 3113 livestock and dogs in 2 climatic seasons (rainy and dry seasons) were tested for MARV antibodies using an indirect ELISA test. The samples were further tested using dot blotting to substantiate the presence of antibodies against MARV glycoprotein (GP). Overall, MARV antibodies were detected in 20.6% of the animals. The species-specific prevalence was 28.7% in cattle, 21.8% in sheep, 19.5% in goats, 15.3% in dogs and 11.2% in pigs. The seropositivity was higher in the rainy season [RR 1.5; 95% CI 1.3–1.8] and in older animals [RR = 2.6; 95% CI 1.9–3.4]. The findings underscore the importance of regular surveillance using the one health approach and future studies into the role of livestock and dogs as potential intermediaries in the circulation of MARV.

## 1. Introduction

Marburg virus disease (MVD) is a zoonotic hemorrhagic fever caused by Marburg virus (MARV), which belongs to the family *Filoviridae* and a member of the genus *Orthomarburgvirus marburgense.* [1]. The disease, considered to have a high case fatality rate of up to 88% [2,3], can be transmitted from non-human primates such as monkeys and gorillas to humans [4,5]. The Marburg and Ebola viruses are both members of the *Filoviridae* family [1].

Some species of fruit bats including Egyptian rousettes, *Rousettus aegyptiacus,* are known to be natural reservoirs of MARV [6,7,8]. While the presence of most filoviruses such as the Ebola virus have been described in domesticated animals and livestock [9], it remains unclear whether MARV can infect livestock [10].

The continued expansion of the human population has led to the disruption of animal habitats and frequent interactions between domestic livestock, humans, and reservoir hosts [11]. Food and companion animals, including hunting dogs, can act as bridging hosts for viral transmission between wildlife and humans [12]. This increases the risk of otherwise improbable spill-over events in human populations [13]. In Africa, outbreaks of MVD are associated with high case fatality rates as reported in Angola (90%), Uganda 25%), Kenya (100%), South Africa and Democratic Republic of Congo (83%) [14,15]. The first outbreak of MVD in West Africa was reported in Guinea in 2021 [16]. Ghana recorded its first human case of MVD in June 2022 in the Ashanti region [17]. Although the outbreak was quickly brought under control and declared over by mid-September 2022 with a case fatality rate of 75% [18], the epidemiology and transmission dynamics of the disease in Ghana remain unclear.

The novel occurrence of MVD in Ghana requires an investigation into the source of infection and possible spill-over from known reservoirs. Domestic animals and livestock can serve as intermediaries for the spread of infections including filoviruses from wildlife reservoirs to humans during spillover events [12,19]. Although antibodies to filoviruses have been reported in bats in Ghana [20], information on the possible spillover to domestic animals has not been documented. The aim of this study was to determine the occurrence of MARV antibodies in livestock and dogs in selected regions of Ghana to aid in the development of risk assessment and mitigation strategies with the ultimate goal of preventing future events in this country.

## 2. Materials and Methods

### 2.1. Study Site and Design

The study was conducted in 8 communities drawn from 5 of the 16 administrative regions of Ghana, namely Western (Bogoso), Ashanti (Fomena and Kwaman), Volta (Ve Golokuati), Savannah (Gelenkong and Mognori) and Bono East (Buoyem and Tanoboase) regions. The Bogoso and Fomena communities were selected based on the incidence of MVD in humans in 2022 [21] and the presence of extensive mining and farming by migrants [22]; Gelenkong and Mognori are fringe communities of the Mole National Park; Tanoboase, Buoyem, and Ve Golokuati were chosen for the presence and close association between the inhabitants and fruit bats [23,24]. A cross-sectional study was carried out in two climatic seasons: dry and rainy seasons. The south of Ghana experiences two rainy seasons, April to June and September to November, and a dry season from December to March [25]. The northern part of Ghana experiences a long dry season from November to June [25]. The dry season sampling was conducted in February to March and July to August 2023 for the rainy season.

### 2.2. Sample Size Determination

In the absence of prevalence data of MARV in domestic animals in the West African region, the maximum prevalence of 50% was used as an estimate to calculate the sample size. At a 95% confidence interval, and a maximum allowable error of 5%, the sample size was computed to be 385 animals. The obtained sample size was multiplied by a design effect (D) of 3 using an intra-cluster correlation coefficient (rho) of 0.25 and an average cluster size of 9 based on the formula D = 1 + p (m − 1) [26]. This gave a total sample size of 1155 animals for each season, giving 231 per species per season, and 2310 in total for the study.

### 2.3. Sampling

A total of 5 mls of blood was obtained from the jugular vein of livestock and the cephalic vein of dogs, directly drawn into a 5 mL vacutainer tube with clot activator and gel for serum separation. The samples were stored in liquid nitrogen for onward transportation and testing at the Accra Veterinary Laboratory, Veterinary Services Department of the Ministry of Food and Agriculture. The samples were centrifuged at 2500 rpm for 3 min and the sera aliquoted into cryogenic vials and stored at −20 °C till testing. A structured questionnaire (Supplementary S1) was administered to farmers to obtain demographics, production history, and husbandry practices. The age of the animals was provided by the owners and estimated with dentition where there was doubt. The animals were age-categorized into young (3 to 12 months), young adult (12 months to 24 months), and adults (above 24 months). Animals aged 3 months and below were excluded. The farming system for the livestock was categorized as intensive for animals housed day and night, and semi-intensive for animals penned at night and allowed to roam during the day. The extensive system involved animals that were allowed to roam freely both day and night. The source of animals was considered to be from within the community when purchased locally, and from outside when sourced from outside of the community.

### 2.4. Laboratory Analysis

#### 2.4.1. ELISA

The total specific immunoglobulin gamma (IgG) against Marburg glycoprotein was measured using animal species agnostic, an in-house ELISA protocol. Nunc Maxisorp (Denmark) plates were coated with 50 μL per well of 0.2 μg/mL Marburg glycoprotein, Musoke (IBT catalogue # 0503-001, Rockville, MD, USA) in PBS (pH 7.2) and incubated at 4 °C overnight. The plates were washed four times with phosphate-buffered saline with 0.05% Tween-20 (PBST), 200 μL per well, and blotted dry on tissue paper. Blocking was performed with 3% Bovine Serum Albumin (BSA) prepared in PBST, 200 µL per well, at room temperature for 2 h.

A volume of 50 μL of primary antibodies, in serum samples, were added at a concentration of 1:100 in duplicates. Additionally, a positive control of a pool of high anti-Marburg glycoprotein IgG responders determined from an initial ELISA run was added at a concentration of 1:200. From this, a standard curve was generated by a 2-fold dilution across seven pairs of wells of the pooled high IgG responders for each species. A negative control, a pool of low anti-Marburg IgG responders, was added at a concentration of 1:100. Primary antibody incubation was performed at room temperature for 1 h. After washing four times with PBST, the plates were incubated with a 1:10,000 concentration of protein G (HRP) (Abcam, ab7460, Cambridge, United Kingdom) for 1 h at room temperature. The plates were washed and blotted dry. The color reaction was generated using 100 μL of 1-Step Ultra TMB-ELISA (ThermoScientific, 34029, Rockford, IL, USA) for the cattle, sheep, and dog samples, and KPL ABTS Peroxidase substrate (seracare, 5120-0046, Milford, MA USA) for the pig and goat samples. The solvent used for the % BSA was Tris-buffered saline with 0.1% Tween^®^ 20 detergent (TBST). Incubation was conducted in the dark for 2 min (cattle and sheep), 5 min (dogs), and 15 min (pigs and goats). The color reaction was stopped with 100 μL per well of 0.27N H_2_SO_4_ for the cattle, dog, and sheep samples and 1% SDS for the pig and goat samples. The plates were read using the Varioskan ELISA plate reader at 450 nm wavelength for the cattle, dog, and sheep samples, and 405 nm wavelength for the pig and goat samples.

Arbitrary antibody concentrations were generated from optical densities by four-parameter fitting implemented in the Auditable Data Analysis and Management System for ELISA (ADAMSEL). Seropositive cut-offs were determined to be the mean of the negative controls plus three times the standard deviations of the negative controls. Data were analyzed in R Software for Statistical Analysis version 4.3.1.

#### 2.4.2. Dot Blot

Selected animals that tested positive for MARV antibodies and those that tested negative using ELISA were subjected to dot blot for confirmation. A volume of 5 μL containing 1.4 μg of purified Marburg glycoprotein, Musoke (IBT catalogue # 0503-001, Rockville, MD, USA) was spotted in pairs on a 0.2 μm nitrocellulose membrane. The membrane was allowed to dry at room temperature for 30 min and blocked with 5% bovine serum albumin (BSA) in phosphate-buffered saline containing 0.005% Tween-20 (PBST) for 1 h at room temperature. Afterwards, it was washed four times, 10 min each, on a shaker with PBST. Serum samples that tested positive in ELISA were prepared at a dilution of 1:500 in 1% BSA. Serum samples that tested negative in ELISA were also diluted at similar dilution. A volume of 5 mL of the diluted test serum samples (ELISA-positive and -negative test sera on separate blots) was added to the membrane and incubated at room temperature for 1 h. The membrane was washed four times with PBST. The blot was further treated with Protein G (HRP) (abcam, ab7460, Milford, MA, USA) at a concentration of 1:5000 prepared in 1% BSA for 1 h at room temperature. The membrane was washed four times with PBST. The reaction was generated using Pierce™ ECL Western blotting substrate. Image acquisition was performed using the Amersham™ 600 imager.

### 2.5. Data Analysis

Data were collected using KoboToolbox^®^ app and analyzed using Stata SE version 16 (StataCorp, College Station, TX, USA). We determined seroprevalence for each of the five species tested. The bivariate relationship between MARV and each risk factor was assessed using the Chi-square test of independence (and Fisher’s exact test), followed by a multivariable modified Poisson regression model with reported Odds ratios and their corresponding CI. All statistical analyses were conducted at a 95% confidence level.

## 3. Results

**Figure 1:**
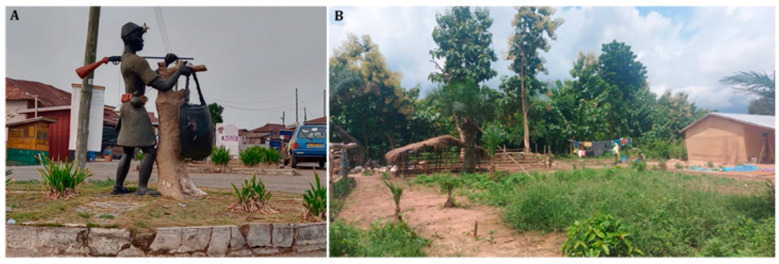
(**A**) picture showing the entrance of a study site, Ve-Golokauti depicting the importance of hunting to its inhabitants. (**B**) shows a residence in the forests of Kwaman depicting the closeness of human settlements to the forests.

A total of 3113 animals were tested from 5 regions of Ghana; 55.4% (1726) and 44.6% (1387) in the dry and rainy seasons, respectively. The proportions of animal species tested were dogs (25.2%), pigs (23.6%), goats (17.6%), sheep (16.9%), and cattle (16.6%). Altogether, the animals were females (65.9%), adults (54.7%), young adults (24.1%), and young (21.2%) and drawn from extensive (43.1%), semi-intensive (42.2%), and intensive (14.7%) production systems. Hunting was practiced in most of the communities sampled (Figure 1).

### 3.1. Prevalence of MARV in Livestock and Dogs

**Figure 2:**
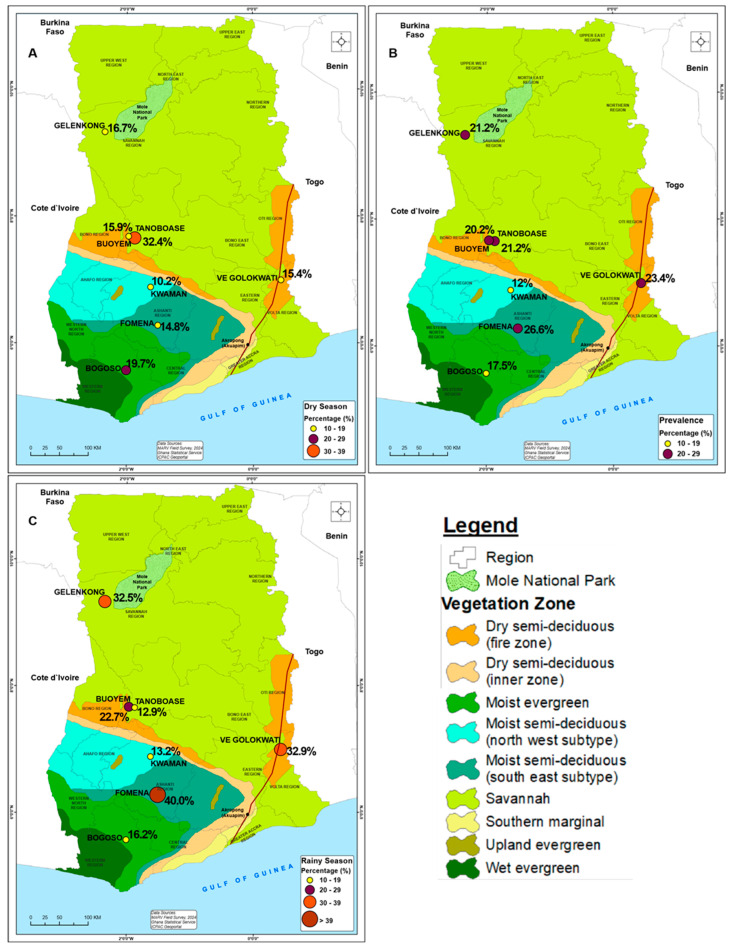
Map of Ghana showing the study locations with prevalence of MARV GP-specific antibodies, 2023. (**A**–**C**) The locations of the study site in relation to known habitats of *Rousettus aegyptiacus* in Ghana [24]. Gelenkong is a community adjacent to the Mole National Park which has a dense population of wildlife including fruit bats, ungulates, and non-human primates. Fomena, Kwaman and Bogoso have dense forest and cave systems. They have high mineral deposits which attracts miners from neighboring regions and countries. The index case of MVD in Ghana was recorded in the Bogoso and Fomena enclave [17,27]. Buoyem and Tanoboase are part of the Mampong range while Ve Golokwati is located along the Akuapim–Togo range. The Akwapim–Togo range has intricate cave systems and extensive forests home to several species of fruit bats including the *Rousettus aegyptiacus.* Generally, the forests serve as pasture for livestock and hunting grounds for hunters who send hunting dogs to capture wildlife. In Ve Golokwati, the dogs in the community serve as hunting dogs irrespective of their original purposes. The dogs may also be borrowed by other hunters for hunting purposes.

**Figure 3:**
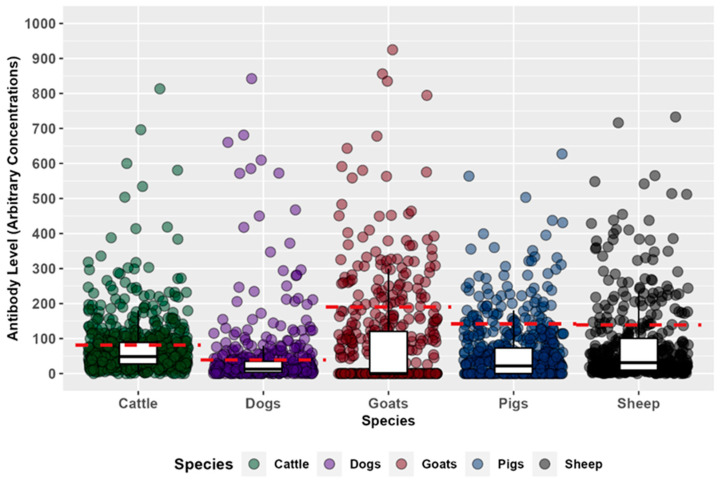
Distribution of anti-Marburg glycoprotein IgG levels in cattle, dogs, goats, pigs, and sheep. Antibody distributions are shown as boxplots underlaid with dots representing individual samples. Boxplots are represented as lower quartile, median, and upper quartile; whiskers are 1.5 times the interquartile range. The red dashed lines represent the seropositive cut-off point for each species. The seropositive cut-offs are as follows: cattle (81.21), dogs (39.43), goats (138.99), pigs (141.93), and sheep (138.99). Seropositive samples are samples with anti-Marburg glycoprotein IgG levels greater than the seropositive cut-off.

The distribution of MARV seropositivity is presented in Table 1, Figure 2 and Figure 3. The overall prevalence of MARV antibodies in the animals was 20.6%. At the regional level, the prevalence was highest in the Savannah region (26.0%) and during the rainy season (32.9%) than the dry season for the remaining four regions. However, Bono East (22.5%) recorded the highest proportion of animals with MARV antibodies during the dry season compared to the other regions.

Young animals (11.6%) exhibited lower seroprevalence compared to the young adults (25.4%) and adults (22.1%). The season of the year (dry and rainy), region, species, age, and source of animals were found to be associated (*p* = 0.001) with the prevalence of MARV GP-specific antibodies in the animals. In comparing species within the region pigs (63.1%) recorded the highest prevalence in the Savannah region followed by cattle (57.9%) in Ashanti, goats (37.5%) in Bono East, dogs (30.7%) in Volta, and sheep (26.2%) in the Western region.

The community-level prevalence of MARV GP-specific antibodies in the species is presented in Table 2. The prevalence was highest in cattle in Kwaman, dogs in Ve Golokuati, goats in Buoyem, Pigs in Gelenkong, and sheep in Tanoboase.

Altogether at the community level, Fomena (40.0%) and Tanoboase (32.4%) recorded the highest proportion of animals with MARV GP-specific antibodies in the rainy and dry seasons, respectively (Figure 2). However, Ve Golokauti (15.4% in the dry season and 32.9% in the rainy season) had relatively high antibody prevalence in both seasons while Mognori had no animals with MARV GP-specific antibodies.

To substantiate the ELISA results, we performed dot blot analysis on selected ELISA-positive and -negative serum samples from the various species (Figure 3). The dot blot results confirmed the positive reactivity of the ELISA-positive sera and the negative results of the ELISA-negative sera (see Supplementary Figures S2–S7).

#### Species-Specific Prevalence

The seroprevalence was highest in cattle during the rainy season compared to other species. In the dry season, sheep recorded the highest prevalence followed by dogs. Animals obtained from mixed sources (sources other than from within the community where they live) had higher seroprevalence (26.1%) than those obtained from within (19.7%) the community (*p* = 0.03).

Assessing the risk factors, the odds of the animals being seropositive for MARV antibodies were 1.7 and 1.4 times higher in Savannah and Volta, respectively, compared to the other regions (Table 3).

## 4. Discussion

In this study, we report the first-time detection of MARV GP-specific antibodies in livestock and dogs from eight communities in five regions in Ghana. Our findings suggest the probable involvement of livestock and companion animals in the epidemiology of the virus. While antibody positivity in ELISA may be prone to potential cross-reactivity, further substantiation of the positive and negative sera in dot blot analysis suggests that domestic animals in the study regions may be exposed to a potential MARV circulation in the domestic–wildlife interface. This requires further investigation.

**Figure 4:**
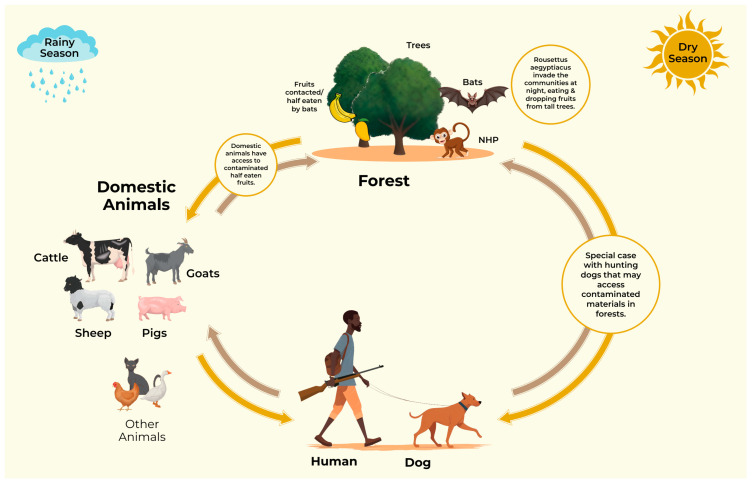
A diagrammatic depiction of the interaction between domestic animals and wildlife which may have led to exposure to MARV. The hunters go to the forest with hunting dogs to capture rodents and other wildlife. The dogs may also pick up dead bats or fruits partially eaten by bats on the forest bed. The domestic animals interact with wildlife as they stray into forests to pasture [46]. These animals may be exposed to MARV and other filoviruses through the consumption of fruit that has been partially eaten by bats [39] and direct interaction with bats. Illustration by Kweku Aboagye, Ghana.

MARV GP-specific antibodies were recorded in all seven communities in the five regions studied. The results suggest the circulation and possible endemicity of MARV in the study sites and we have sought to schematically depict (Figure 4) what we gather as some ways in which the animals could have been exposed to MARV. Fruit bats in Ghana are densely distributed along what is known as the Akwapim–Togo range (Figure 2) [28,29]. The Akwapim–Togo range is a narrow belt of ridges and hills in Ghana (and partly Togo) noted not only for habitats for the Egyptian fruit bats but caves and niches in trees in its immediate environments [24,30]. In recent years, the expansion of the range has been associated with mining activities, deep-forest cocoa farming, and deforestation which could lead to increased interaction between wildlife, livestock, and humans. The question as to whether the presence of MARV GP-specific antibodies detected in this study areas could be due to this interaction is yet to be determined and requires further investigation. Given the role livestock and dogs play in the spread of zoonotic diseases [12,31,32,33], particularly in communities where the wildlife–dog–livestock interface is established by cultural and farming practices in Ghana [23], the role of livestock and dogs in the transmission dynamics of MARV needs to be further studied.

The identification of orthoebolavirus antibodies in pigs in Guinea [34], Sierra Leone [35], and Ghana [36], and the high levels of MARV antibodies in apparently healthy livestock and dogs in this study is suggestive of the relevance of domestic animals as potential epidemiological links in the spread or maintenance of filoviruses in at-risk geographical areas like Ghana [17,27]. This underscores the need for implementing active surveillance for early detection of emerging zoonotic pathogens in at-risk domestic livestock species and enhancing preparedness for emerging and re-emerging disease outbreaks [37,38].

At the community level, the animals in Fomena, Ve-Golokauti, Tanoboase, Gelenkong and Buoyem recorded the highest MARV GP-specific antibodies. Fomena has several mountains and forests with cave systems where migratory cocoa farmers and miners in the informal economic sector congregate to undertake their activities. The inhabitants of Fomena have a close relationship with wildlife, with some livestock feeding on fallen fruit, including fruits that are partially eaten by bats. The scavenging of leftover fruits, which may be contaminated with infected bat urine or saliva, could increase the animals’ exposure to MARV-infected bats as described in Bangladesh [39].

Ve-Golokuati is a culturally rich hunting community with close interaction with fruit bats [23], livestock, and dogs in the Volta Region. This district also falls within the Akwapim–Togo range. Buoyem and Tanoboase are small communities enclosed by sandstone hills, forests, and caves famed for the presence of the Egyptian fruit bats [28,40]. Gelekong in the Savannah region is located at the outskirt of the Mole National Park (Figure 4) which is home to several fruit bats and other wildlife [41], as well as a major livestock market. The inhabitants and livestock in these communities have close interaction with wildlife due to its proximity to the park, encroachment of land and expansion of human settlement.

The proportion of MARV GP-specific antibodies in livestock and dogs was found to be higher in the rainy season compared to the dry season, altogether. Outbreaks of MVD have been linked to the breeding seasonality of Egyptian fruit bats with juvenile bats implicated in spillover events [7]. In Ghana, the breeding patterns of fruit bats appear to favor a high population of juvenile bats during the rainy season [24], though it is not certain if this contributed to the higher prevalence in the rainy season. Additionally, the integration of crop farms in forest areas with *Cedrela odorata* (Spanish-cedar) plant [42,43] provides food sources for bats during the rainy season [24]. Transmissions of infections in West Africa have occurred through the exposure of humans and livestock to fruits partially eaten by fruit bats [39,44]. These conditions could provide grounds for increased interactions between livestock, dogs, and wildlife and is exacerbated by the fluid nature of the human–wildlife relationships in the study sites.

The prevalence of MARV antibodies was found to be higher in dogs in the Ve Golokuati in the Volta region where hunting is considered a part of life [45,46]. Hunting peaks at the end of the dry season and the beginning of the rainy season, when farmers burn pasture or forests to allow for the rapid growth of plant shoots with the first rains [47]. Therefore, the observed increase in the MARV GP antibodies in dogs in the dry season could be attributed to increased contact with wildlife a result of this phenomenon.

It is uncertain why cattle showed the highest MARV GP antibodies compared to the other species sampled. However, in the dry season, adult ruminants are normally sent over long distances in search of pasture, leaving behind the younger animals to feed on shrubs in the community; this may be increasing their likely exposure to infectious pathogens like MARV.

The prevalence of MARV antibodies in pigs in Gelekong was the highest recorded for pigs in this study. In Gelenkong, pigs are left to scavenge for food (extensive production system) with temporary structures provided during rainy seasons. The pigs tend to wander into wildlife zones and that could increase their risk of exposure to diseases.

The source of livestock showed an association with the presence of MARV antibodies in the study sites. Animals from mixed sources showed high exposure rates. Farmers within these communities do not quarantine or examine newly introduced animals prior to adding them to existing stock. This could increase the risk of introduction of pathogens from external sources.

## 5. Conclusions

We report the first detection of MARV GP-specific antibodies in livestock and dogs from seven communities in five regions of Ghana. Communities in Savannah and Volta regions recorded the highest prevalence compared to the other communities. The season of the year, region, species, age, and source of animals were found to be associated with the prevalence of MARV in the animals. Therefore, these findings provide a basis for further studies into the role of livestock and other domestic animals in the epidemiology of MARV. The results also underscore the need for a coordinated, multifaceted research, and serve as a wake-up call for investment in developing an early warning system and preparedness in the West African subregion.

## Figures and Tables

**Table 1:** Prevalence and bivariate analysis of MARV GP-specific antibodies in selected regions of Ghana, 2023.

Variable	N	Prev (%)	*p*	Dry Season			Rainy Season		
				N	Prev (%)	*p*	N	Prev (%)	*p* Value
All species	3113	20.6		1386	16.8	0.001	1725	23.7	
Region	3113		0.002			0.015			0.001 *
Ashanti	691	17.7		309	12.3		382	21.9	
Bono East	627	20.6		240	22.5		387	19.4	
Savannah	323	26.0		133	16.7		190	32.8	
Volta	839	23.4		456	15.4		383	32.9	
Western	633	17.5		249	19.7		384	16.2	
Species	3113		0.001			0.001			0.001 *
Dogs	785	15.3		374	19.8		411	10.9	
Goats	548	19.5		304	12.8		244	28.7	
Sheep	526	21.8		303	22.4		223	20.2	
Cattle	521	28.7		142	9.9		379	34.1	
Pigs	733	11.2		264	14.4		469	25.6	
Type of production	3029		0.513			0.371			0.121
Extensive	561	21.9		287	15.7		274	28.8	
Semi-intensive	1812	21.1		768	18.4		1044	23.1	
Intensive	856	19.4		256	15.1		398	22.1	
Age	3105		0.001			0.003			0.001 *
Young	658	11.6			291	10.7	367	12.3	
Young adults	749	25.4			400	20.3	349	31.2	
Adults	1698	22.1		687	17.5		1011	25.2	
Sex	2881		0.512			0.167			0.901
Female	1901	21.4		805	18.0		1096	23.8	
Male	980	20.3		360	14.7		620	23.6	
Source of Animals	3029		0.030			0.007			0.001 *
Mixed source	2183	19.7		1055	18.5		1128	20.9	
Outside community	616	22.9		233	10.3		383	30.6	
Within same community	230	26.1		25	24.0		205	26.3	

MARV (Marburg virus). GP—Glycoprotein Prev: prevalence. Differences in N are due to missing data during questionnaire administration. *p* value < 0.05 is significant (*). Species, age, and sources of animals were associated with the prevalence of MARV antibodies in the animals.

**Table 2:** Bivariate analysis of species-specific MARV GP-specific antibodies in communities in five regions of Ghana.

Species	Bogoso		Buoyem		Fomena		Gelenkong		Kwaman		Tanoboase		Ve ^1^ Golo		*p* Value
	n	%	n	%	n	%	n	%	n	%	n	%	n	%	
Cattle	135	21.5	40	47.5	86	56.7	0	0	9	66.6	53	20.7	196	14.7	0.001 *
Dogs	166	15.1	103	13.6	38	5.3	61	8.2	150	6.0	78	7.9	189	41.2	0.001 *
Goat	118	9.3	80	37.5	28	25.0	101	22.7	78	17.9	0	0	143	16.7	0.001 *
Pigs	130	18.5	138	9.4	84	5.9	65	63.1	109	2.7	19	52.6	188	32.9	0.001 *
Sheep	84	26.1	40	12.5	31	25.8	94	16.0	78	24.3	76	27.6	123	18.7	0.260

%: species—specific prevalence. ^1^ Ve Golokuati. *p* value < 0.05 is significant (*).

**Table 3:** Multivariable analysis of factors associated with MARV GP-specific antibodies in livestock and domestic dogs, Ghana.

Parameter	Prevalence Ratio [95% CI]	*p*-Value
Region		
Ashanti	1 *	
Bono East	1.2 [0.8–1.5]	0.243
Savannah	1.7 [1.2–2.3]	0.002
Volta	1.4 [1.1–1.8]	0.007
Western	0.9 [0.7–1.2]	0.714
Species		
Cattle	1 *	
Dogs	0.5 [0.3–0.6]	0.001
Goat	0.7 [0.5–0.9]	0.013
Pigs	0.7 [0.5–0.9]	0.021
Sheep	0.8 [0.5–1.0]	0.073
Age		
Young	1 *	
Adults	2.2 [1.6–2.8]	0.001
Young adults	2.6 [1.9–3.4]	0.001
Source of animals		
Mixed source	1 *	
Outside community	0.8 [0.5–1.1]	0.331
Within same community	0.6 [0.5–0.9]	0.024
Season of the year		
Dry season	1 *	
Rainy season	1.5 [1.3–1.8]	0.001

* represents the base variable.

## Data Availability

The raw data supporting the conclusions of this article will be made available by the authors on request.

## Supplementary Materials

The following supporting information can be downloaded at https://www.mdpi.com/article/10.3390/pathogens13110917/s1, S1: Study Questionnaire; Figure S2: Dot blot image of a negative sample; Figure S3: Dot blot image of a MARV positive cattle sample; Figure S4: Dot blot image of a MARV positive dog sample; Figure S5: Dot blot image of a MARV positive goat sample; Figure S6: Dot blot image of a MARV positive pig sample; Figure S7: Dot blot image of a MARV positive sheep sample.
