# Supplementary material for: Serological Evidence of Potential Marburg Virus Circulation in Livestock and Dogs in Ghana
Source: Pathogens. 2024 Oct 22;13(11):917. doi: 10.3390/pathogens13110917 (PMC11597086; doi:10.3390/pathogens13110917)
Supplement: Supplementary file 1 [file pathogens-13-00917-s001.zip › Questionnaire_Marburg_Supplementary Figure S1.pdf]

QUESTIONNAIRE-Global Partnership for Animal Zoonotic Disease Surveillance (GPAZDS)

MARBURG INVESTIGATION IN COMMUNITIES AT RISK IN GHANA

We are a team of researchers interested to investigate a group of viruses that affect humans and animals called Filoviruses. Our focus is on one of them named Marburg virus which was recorded in parts of Ghana some months ago. Our study is to identify risk factors that predispose humans and animals in the communities to Marburg disease.

We therefore crave your indulgence to ask a few questions, take blood, nasal and rectal swabs from your livestock. There are no known risks to participating in the survey, and any information we obtain from you will be kept confidential. We will inform you of any findings from the livestock samples. There are no payments in participating in this study, however, we have brought dewormers to use on your livestock if you do not mind. Your participation is voluntary, and there are no consequences if you decide not to participate. The survey asks about your background, household, animals, and their contacts with other animals. It will take about 20 minutes, and you can skip any questions that you do not want to answer and stop at any time. Trained veterinarians will take samples from your livestock with no or minimal pain experienced.

This project has been approved by the ……, and your Assembly, Chiefs, and opinion leaders have been notified about our work.

Do you have any questions?

*Do you agree to participate in this survey?

- Yes
- No

DRAFT

Objectives:

- Socio-ecological conditions
- Epidemiological (location GIS, place, time)
- Behavioral
- Movement of livestock (data review)

Risk factors: People, Animal, Wild and Environment

DATE: ……………………

UNIQUE ID: ……………………….. GPS: ……………………………

Community:……………………….. District:…………………….. Region: ……………………..

QUESTIONNAIRE-Global Partnership for Animal Zoonotic Disease Surveillance (GPAZDS)

Interviewer: ………………………………………………………..……

SECTION A: SOCIO-DEMOGRAPHIC DETAILS OF RESPONDENTS

1. Gender
- Female
- Male

2. Age
- <10years
- 10-20years
- 21-30years
- 31-40years
- 41-50years
- >50years

3. Highest level of education
- Basic level
- Secondary level
- Tertiary level
- None
- Other….. (including non-formal and Arabic education)

4. Occupation
    - Crop farmer
    - Livestock farmer
    - Petty trader
    - Other ……………………………

5. Religion
- Islam
- Christian
- Traditional
- Other………….

DRAFT

6. Current employment status
   - Employed
   - Unemployed

7. For respondents who are employed ask:
   Source of income (select all that may apply)
   - Crop production
   - Animal production
   - Meat processing (khebab seller, meat seller, chop bar operator)
   - Slaughter house/abattoir
   - Bushmeat trader
   - Hunter
   - Trapper
   - Fishing
   - Forest product collector (firewood, mushroom, traditional healers)
   - Mining
   - Bat dropping (guano) collection (from caves)
   - Construction
   - Migrant laborer
   - Other …….

8. For unemployed respondents ask:
   Livelihood sources
   - Remittances
   - Other………

9. How many people currently live in your home, including yourself?
   - 1-3
   - 4-6
   - 7 and above

Section B: Ownership and or access, production and management of livestock

10. Ownership
    - Owner
    - Caretaker
    - Other …………………….

11. How long have you been keeping livestock for? ………….

DRAFT

- <1year
- 1-3years
- 4-6years
- 7 and above

12. Do other people have livestock in the same household?
- Yes
- No

13. What types of animals do you (and other household members) keep?
- Small ruminants (Sheep, goat)
- Cattle
- Pigs
- Poultry (local fowls, commercial fowl)
- Dogs
- Cat
- Donkey
- Other …………………….

14. What are the source of the animals in your household?
- Within community
- Outside community
- If outside state where ……………………
- Other………

15. Are the animals housed?
- Yes
- No

16. Type of production system
- Intensive
- Semi-intensive
- Extensive

17. Do any of your animals have contact with wild animals.
- Yes
- No
- I don't know

DRAFT

18. How are your livestock kept at night.

- In a pen
- Open field
- Both
- Other…

19. Are new livestock quarantined before adding to the old stock.

- Yes
- No
- Unsure

20. How do you dispose animal waste?

- Use as manure
- Throw away
- Other …………………..

21. When you domestically slaughter/kill animals, what happens to the viscera (blood, organs, skin etc).

- Sell
- Throw into refuse bin
- Throw into the street/gutter
- Feed to animals
- No onsite slaughter
- Other…….

Section C: Contact with other animals (including wildlife)

22. Do any of your animals have contact with the following animals?
(Select all that apply)

- Rodents
- Bat
- Monkey
- Antelopes
- Other ………………………………
- No
- I don't know

23. Do you find bats in your household?

- Yes

DRAFT

- No

24. Have you seen bats flying overheard?
- Yes
- No
- I don't know

25. Do your livestock eat fruits that have fallen on the ground?
- Yes
- No
- I don't know

26. Have you seen any groups of dead animals of the same kind (as yours)? (Not intentionally killed by humans and not meant for markets).
- Yes
- No
- I don't know

27. What do you do when you find your animal dead?
- Avoid it
- Dispose of/ bury it
- Eat
- Sell the meat
- Share the meat
- Feed it to domestic animal
- Other………………..

28. What are the commonest disease signs that your animals experience?
    - Respiratory
    - Digestive
    - Skin
    - Musculo-skeletal
    - Other………………..

Water source

29. What is your source of drinking water? (select all that apply)
- Pipe borne water
- Borehole
- Outdoor piped water

- Stream or river
- Pond or lake or dam
- Other ………………………

30. Do you have containers for storing drinking water for the household?
- Yes, with covers
- Yes, without covers
- No
- Don't know/ prefer not to say

Toilet facilities

31. Type of toilet facility in the household.
- Home water closet
- Home latrine
- Public WC
- Public toilet
- Free range (open defaecation)

32. Other factors

How do you usually store left-over food in your household overnight? (Select all that apply)
- Sealed, covered containers
- Unsealed, covered containers
- Uncovered containers
- Not in a container
- Don't know/ prefer not to say

Thank you for participating in this study.

DRAFT

QUESTIONNAIRE-Global Partnership for Animal Zoonotic Disease Surveillance (GPAZDS)

SAMPLING

No	Animal ID	Specie	*Age	Sex	Breed	Clinical signs (if any)	ELISA result	PCR result
1								
2								
3								
4								
5								
6								
7								
8								
9								

*in the absence of an estimated age, they can be qualified as young, adults

DRAFT
