# Supplementary figures and images for: Serological Evidence of Potential Marburg Virus Circulation in Livestock and Dogs in Ghana
Source: Pathogens. 2024 Oct 22;13(11):917. doi: 10.3390/pathogens13110917 (PMC11597086; doi:10.3390/pathogens13110917)

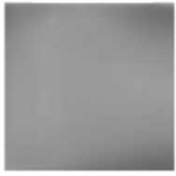

Supplement: Supplementary file 1 [file pathogens-13-00917-s001.zip › Negative sample_Supplementary Figure S2.png]

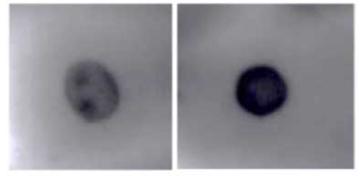

Supplement: Supplementary file 1 [file pathogens-13-00917-s001.zip › positive cattle sample_Supplentary Figure S3.png]

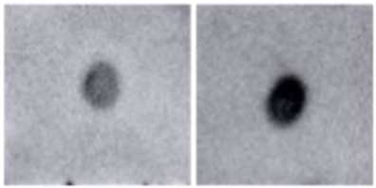

Supplement: Supplementary file 1 [file pathogens-13-00917-s001.zip › positive dog sample_Supplmenatry Figure S4.png]

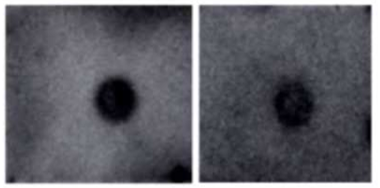

Supplement: Supplementary file 1 [file pathogens-13-00917-s001.zip › positive goat sample_Supplematary Figure S5.png]

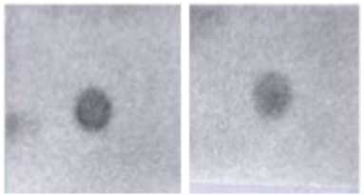

Supplement: Supplementary file 1 [file pathogens-13-00917-s001.zip › positive pig sample_Supplemenatry Figure S6.png]

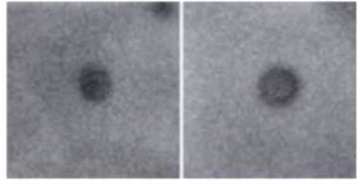

Supplement: Supplementary file 1 [file pathogens-13-00917-s001.zip › positive sheep sample_Supplementary Figure S7.png]
